# Aluminum or Low pH – Which Is the Bigger Enemy of Barley? Transcriptome Analysis of Barley Root Meristem Under Al and Low pH Stress

**DOI:** 10.3389/fgene.2021.675260

**Published:** 2021-05-19

**Authors:** Miriam Szurman-Zubrzycka, Karolina Chwiałkowska, Magdalena Niemira, Mirosław Kwaśniewski, Małgorzata Nawrot, Monika Gajecka, Paul B. Larsen, Iwona Szarejko

**Affiliations:** ^1^Institute of Biology, Biotechnology and Environmental Protection, Faculty of Natural Sciences, University of Silesia in Katowice, Katowice, Poland; ^2^Centre for Bioinformatics and Data Analysis, Medical University of Bialystok, Bialystok, Poland; ^3^Clinical Research Centre, Medical University of Bialystok, Bialystok, Poland; ^4^Department of Biochemistry, University of California, Riverside, Riverside, CA, United States

**Keywords:** barley, RNA-Seq, transcriptome, low pH, aluminum (Al), stress, root meristem

## Abstract

Aluminum (Al) toxicity is considered to be the most harmful abiotic stress in acidic soils that today comprise more than 50% of the world’s arable lands. Barley belongs to a group of crops that are most sensitive to Al in low pH soils. We present the RNA-seq analysis of root meristems of barley seedlings grown in hydroponics at optimal pH (6.0), low pH (4.0), and low pH with Al (10 μM of bioavailable Al^3+^ ions). Two independent experiments were conducted: with short-term (24 h) and long-term (7 days) Al treatment. In the short-term experiment, more genes were differentially expressed (DEGs) between root meristems grown at pH = 6.0 and pH = 4.0, than between those grown at pH = 4.0 with and without Al treatment. The genes upregulated by low pH were associated mainly with response to oxidative stress, cell wall organization, and iron ion binding. Among genes upregulated by Al, overrepresented were those related to response to stress condition and calcium ion binding. In the long-term experiment, the number of DEGs between hydroponics at pH = 4.0 and 6.0 were lower than in the short-term experiment, which suggests that plants partially adapted to the low pH. Interestingly, 7 days Al treatment caused massive changes in the transcriptome profile. Over 4,000 genes were upregulated and almost 2,000 genes were downregulated by long-term Al stress. These DEGs were related to stress response, cell wall development and metal ion transport. Based on our results we can assume that both, Al^3+^ ions and low pH are harmful to barley plants. Additionally, we phenotyped the root system of barley seedlings grown in the same hydroponic conditions for 7 days at pH = 6.0, pH = 4.0, and pH = 4.0 with Al. The results correspond to transcriptomic data and show that low pH itself is a stress factor that causes a significant reduction of root growth and the addition of aluminum further increases this reduction. It should be noted that in acidic arable lands, plants are exposed simultaneously to both of these stresses. The presented transcriptome analysis may help to find potential targets for breeding barley plants that are more tolerant to such conditions.

## Introduction

One of the biggest problems of modern agronomy and a constraint for world agriculture is the progressive acidification of arable lands, caused by industrial pollution and overuse of ammonia- and amide-containing fertilizers. It is estimated that up to 50% of arable lands worldwide are acidic, with a pH below 5.5 (Von Uexküll and Mutert, 1995; Singh et al., 2017; Barros et al., 2020). The majority of crops growing in acidic soils show significant yield losses - up to 80%, depending on the species (Sade et al., 2016). The primary factor responsible for reduced yield in acidic soils is aluminum (Al), the third most abundant element (after oxygen and silicon) and the most common metal in the Earth’s crust. In alkaline and near-neutral soils, Al is bound in various minerals or occurs in forms that are mostly harmless to plants. However, in acidic soils, Al is released from clay minerals in the form of [Al(H_2_O)_6_]^3+^, for simplicity often referred to as Al^3+^ ions, that are bioavailable for plants and highly phytotoxic (Bhalerao and Prabhu, 2013; Sade et al., 2016; Rahman et al., 2018).

The first symptom of Al toxicity in acidic soils is reduction of root growth, resulting from inhibition of both elongation and division rates of root cells. As a consequence, the plant suffers from reduced water and nutrient uptake, which leads to plant growth retardation and, finally, yield reduction. It has been shown that Al^3+^ ions are highly reactive and there are many potential Al binding sites in plant cells. Al^3+^ ions interact with the cell wall, cell membrane, and symplastic components; therefore they interfere with a broad spectrum of physical and cellular processes (Kochian et al., 2005, 2015). The first structure in roots that Al^3+^ ions interact with is the apoplast. Aluminum ions directly cross-link the negatively charged carboxyl groups of pectins in the cell wall, which leads to its stiffening and inhibition of cell elongation (Kopittke et al., 2015). A significant part of absorbed Al (30–90%) is accumulated in the apoplast (Silva, 2012; Gupta et al., 2013). Al^3+^ ions interact also with the negatively charged surface of the plasmalemma and displace other ions like Ca^2+^ from phospholipid head groups, which destabilizes the cell membrane and alters its fluidity. It also leads to depolarization of the plasmalemma, which affects cellular ion homeostasis. Additionally, the replacement of Ca^2+^ by Al^3+^ in the plasma membrane increases Ca^2+^ content in the apoplast and therefore stimulates callose deposition. Accumulation of callose inhibits intercellular transport through plasmodesmata (Kochian et al., 2005).

A fraction of Al^3+^ that enters the cytosol may interact with cytoskeletal elements and disturb its dynamics directly or indirectly through modification of e.g., Ca^2+^ signaling cascade. The disturbances in spatial orientation of the cytoskeleton may affect cell expansion and lead to morphological changes and distortion of roots (Sade et al., 2016). Moreover, there is extensive evidence that Al^3+^ ions enter the nucleus, cause DNA damage (Silva et al., 2000; Min et al., 2009; Jaskowiak et al., 2018), and activate the DDR (DNA Damage Response) pathway, which additionally leads to inhibition of cell divisions (Rounds and Larsen, 2008; Nezames et al., 2012; Szurman-Zubrzycka et al., 2019). Furthermore, exposure to Al induces oxidative stress. It promotes the overproduction of Reactive Oxygen Species (ROS) and alters the activity of enzymes responsible for maintaining ROS homeostasis in cells, such as superoxide dismutase and ascorbate peroxidase (Yamamoto et al., 2003; Guo et al., 2004; Jones et al., 2006). The Al-induced overproduction of ROS leads to the peroxidation of lipids and proteins and further DNA damage (Achary and Panda, 2009).

In general, plants evolved two main strategies to cope with Al ions: (1) Al exclusion mechanisms and (2) Al tolerance mechanisms. The first one is based on the production of organic acids (OAs) and their exudation outside the cell. The OAs, such as citric and malic acids, chelate Al in the rhizosphere which prevents its entrance to the root cells. The second strategy deals with Al that entered the cell. The internal OAs and other organic compounds form Al-complexes that are detoxified in vacuoles or reallocated to the upper, less Al-sensitive parts of the plant (reviewed in Kochian et al., 2015; Riaz et al., 2018).

Taken together Al induces a broad spectrum of changes and responses in plant cells. Al stress is considered as the main growth-limiting factor in acidic soils and the second, after drought, most serious abiotic stress to crop production worldwide (Kochian et al., 2015). Barley (*Hordeum vulgare* L.), which is the 4th most important cereal crop, is known to be one of the most sensitive to Al cereal species (Ishikawa et al., 2000; Wang et al., 2006), but its response to Al has not been studied at the whole transcriptome level. Besides, our preliminary studies have shown that barley is very sensitive not only to phytotoxic Al^3+^ ions in acidic conditions, but also to the low pH of growth medium alone. The low pH causes so called H^+^ or proton toxicity. In naturally occurring acidic arable lands, plants are exposed simultaneously to both of these stressors (low pH and Al), as Al becomes soluble at pH below 5.5. However, growing plants in the hydroponic solution makes it possible to examine at the gene expression level the plant response to the stress triggered by low pH without Al, and to reveal changes caused by Al toxicity itself.

Here we show, for the first time, the global transcriptome profile of barley root tips grown in hydroponics at the optimal pH (6.0), low pH (4.0), and low pH with Al (10 μM of bioavailable Al^3+^ ions) in two independent, short-term and long-term, experiments.

## Materials and Methods

### Plant Material

The spring barley (*Hordeum vulgare* L.) cultivar ‘Sebastian’ bred by the Danish company Sejet Plantbreeding was used as plant material in the presented study. This cultivar is a parent variety of barley TILLING population (*Hor*TILLUS) that was developed at the Department of Genetics, University of Silesia in Katowice (Szurman-Zubrzycka et al., 2018) and is extensively used in functional genomics studies.

### Examination of Root Parameters of Barley Seedlings Grown at Low pH and Treated With Aluminum

#### Hydroponic Experiment

The low pH and aluminum treatments were performed in a hydroponic environment as described previously (Szurman-Zubrzycka et al., 2019). Briefly, seeds of barley cv. ‘Sebastian’ were surface-sterilized in 5% sodium hypochlorite and incubated in the dark at 4°C for stratification. Then the seeds were put on Petri dishes filled with moist filter paper and placed in a growth chamber at 25°C in the dark. After 48 h, the germinated seeds were transferred to 4.5 L hydroponic containers with Magnavaca solution (Magnavaca et al., 1987) at pH = 6.0, pH = 4.0, or pH = 4.0 with 10 μM of bioavailable Al^3+^ ions. The concentration of 10 μM of bioavailable Al^3+^ ions was calculated with GEOCHEM-EZ software (Shaff et al., 2010) and it corresponds to 50 μM of nominal AlCl_3_ added to the Magnavaca medium at pH = 4.0. The maximum of 12 seedlings were placed in one container that was considered as one replicate and each experimental combination was set up as three replicates. The seedlings were grown in hydroponics for 7 days (7 d) under controlled conditions: 20°C/18°C (day/night), 16/8 h photoperiod, 250 μM m^–2^ s^–1^ light intensity.

#### Root System Scanning and Analysis

After 7 days, the seedlings were removed from containers and their roots were preserved in 50% ethanol and scanned in water in waterproof trays. For scanning, the EPSON PERFECTION V700 PHOTO scanner with a dual-lens system was used accompanied by WinRHIZO software (Regent Instruments). The root parameters were calculated, based on the obtained scans, with the use of WinRHIZO and SmartRoot^1^ software. Statistical analyses were performed using ANOVA (*P* < 0.05) followed by Tukey’s Honest Significant Difference test (Tukey HSD test, *P* < 0.05).

### Analysis of Root Meristem Transcriptome of Barley Seedlings Grown at Low pH and Treated With Aluminum

Two independent experiments, short- and long-term, were performed for transcriptome analysis.

#### Short-Term Experiment

**FIGURE 1:**
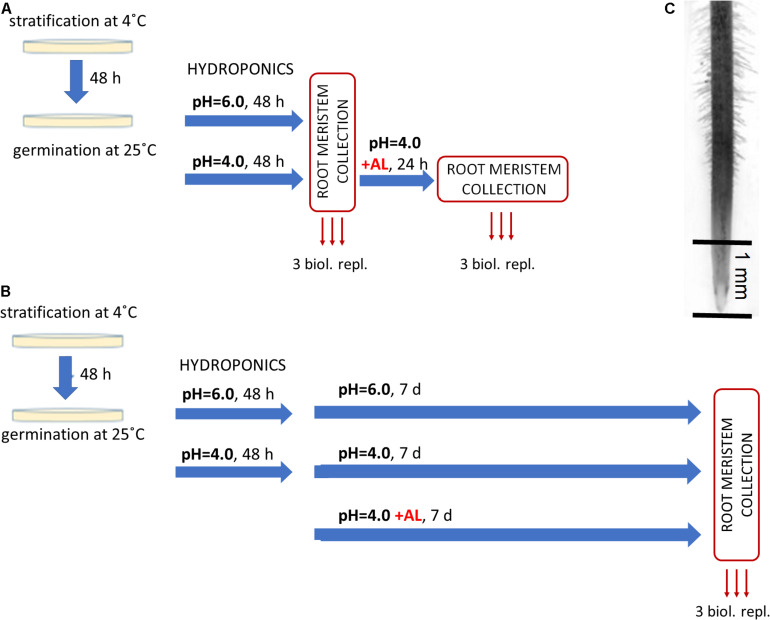
Experimental setup for RNA-Seq analysis. After 48 h of germination on Petri dishes barley seedlings were transferred to hydroponics with Magnavaca medium adjusted to pH = 6.0 or pH = 4.0. After another 48 h AlCl_3_ was added to the medium (10 μM of bioavailable Al^3+^ ions). **(A)** Short-term Al treatment. Root meristems were taken for RNA isolation just before Al addition (as control at pH = 6.0 and pH = 4.0) and after 24 h of Al treatment. **(B)** Long-term Al treatment. Root meristems were taken for RNA isolation from seedlings 7 days after Al addition and from seedlings grown at pH = 6.0 and pH = 4.0 without Al at the same time point. **(C)** The picture of barley root with an indication of its part (root meristem, ∼1 mm of root tip) that was taken for RNA isolation.

The seeds of barley cv. ‘Sebastian’ were germinated as described in section “Hydroponic Experiment.” Germinated seeds were then transferred to 4.5 L hydroponic containers with Magnavaca medium at pH = 6.0 (three containers) and pH = 4.0 without aluminum (six containers). A maximum of 12 seedlings were placed in one container and this was considered as one replicate. After 48 h of seedlings growth, the root meristems (of approximately 1–2 mm length) were collected from three containers with solution at pH = 6.0 and three containers with solution at pH = 4.0, as control samples without Al. Subsequently, the aluminum (10 μM of bioavailable Al^3+^ ions) was added to the remaining three containers with Magnavaca solution at pH = 4.0. After 24 h of Al treatment the root meristems were collected, as Al-treated samples (Figure 1A). The collected root meristems were stored in RNAlater at 4°C for several days for further RNA isolation.

#### Long-Term Experiment

The seeds of barley cv. ‘Sebastian’ were germinated as described in section “Hydroponic Experiment.” Similarly as in the short-term experiment, germinated seeds were transferred to 4.5 L hydroponic containers with Magnavaca solution adjusted to pH = 6.0 (three containers) and pH = 4.0 without aluminum (six containers). After 48 h, 10 μM of bioavailable Al^3+^ ions were added to three containers at pH = 4.0. After further 7 days of seedlings growth, the root meristematic tissue was collected in RNAlater (Invitrogen), as pH = 6.0, pH = 4.0 and Al-treated samples (Figure 1B).

#### RNA Isolation, Preparation of RNA-seq Libraries and Sequencing

For RNAseq analysis, mRNA was isolated from root tips with the use of the Dynabeads mRNA DIRECT Micro Kit (Thermo Fisher Scientific). Root meristems from at least eight plants from one hydroponic container were considered as one repetition (with an average of five root meristems per plant). The RNA-seq libraries were prepared using the TruSeq Stranded mRNA kit (Illumina) according to manufacturer’s instructions. The quality of the prepared RNA-seq libraries was assessed using the TapeStation device (Agilent) and the High Sensitivity DNA ScreenTape kit (Agilent). The concentration of fragments in the libraries was measured with a Qubit fluorimeter (Thermo Fisher Scientific).

For cluster generation, the barcoded libraries were pooled with equimolar concentrations. The libraries from the short-term experiment were sequenced in the paired end (PE) mode 2 × 76 bp, six barcoded samples per lane in the Illumina HiSeq 4000 sequencer at the Genomics and Epigenomics Laboratory, Clinical Research Centre of the Medical University of Bialystok (Poland). The libraries from the long-term experiment were sequenced in the paired end (PE) mode 2 × 150 by the Novogene company (Illumina platform). On average, 59.3 (±14.6) mln reads were obtained per each sample (single biological replicate).

#### Bioinformatic Analysis of RNA-seq Data

BCL files were demultiplexed and converted to fastq files using bcl2fastq (Illumina, San Diego, CA, United States) with an adapters removal step. The quality of the obtained sequencing data was assessed before the analysis and after each of its stages, using the FastQC (The Babraham Institute, Cambridge, United Kingdom) and MultiQC (Ewels et al., 2016) tools. Due to different read lengths in both experiment batches, reads were initially trimmed to the length of 75 bp with BBduk (DOE Joint Genomes Institute, Walnut Creek, CA, United States). Then, quality trimming and filtering was preformed using Sickle tool^2^ under PHRED of 15, N bases removal and minimal length of 20 bp for one mate in the PE mode. The remaining ribosomal RNA reads were then removed using the SortMeRNA software (Kopylova et al., 2012). Filtered non-rRNA reads were mapped to the second version of the reference genome sequence assembly of barley cv. ‘Morex’ (Morex V2; Leibniz Institute of Plant Genetics and Crop Plant Researck – IPK; Monat et al., 2019) with the splice-aware aligner STAR (Dobin et al., 2013) using two pass mode without non-canonical motifs. Mapping parameters were adjusted to the Morex V2 genome annotation from gff3 file provided by IPK, with regard to mates gaps and intron lengths. Only uniquely mapping reads were allowed with maximum 0.05 mismatch rate over read length. The quality of mapping was assessed with QualiMap (Okonechnikov et al., 2016) as well as SAMStat (Lassmann et al., 2011). We applied the high confidence (HC) set of gene annotations in the Morex V2 assembly and counted reads mapping to genes annotated in the gff3 using GeneCounts from the quantMode in STAR mapper. The analysis of differences in gene expression levels between samples was performed with the DESeq2 tool (Love et al., 2014). Raw read count matrices were used as an input and genes without any expression detected were removed. Libraries size factors were estimated using median ratio method and further used in all size normalization steps. Then DESeq function was called on the whole dataset and covered the following steps: sequencing depth normalization between the samples, gene – wise dispersion estimation across all samples, and fitting a negative binomial generalized linear model (GLM) under Wald statistics to each gene. Using a formula with condition factors we applied contrasts for each desired comparison to the results with usage of Cook’s cut-off and independent filtering. Statistical analyzes were performed based on the results obtained from three biological replicates. Differentially Expressed Genes (DEGs) were identified under α = 0.05 after *P*-value correction for multiple comparisons using the Benjamini and Hochberg False Discovery Rate procedure (FDR) and log_2_FoldChange (log_2_FC) ≥ 1 or ≤–1. Exploratory analysis of RNA-Seq data including clustering analysis and Principal Component Analysis (PCA) were carried out with the use of R environment tools. For data inspection and visualization, counts were subjected to regularized logarithm transformation (rlog) to get log2-scaled data that is approximately homoscedastic and normalized with respect to library size. PCA was performed with prcomp function and results were visualized as bi-plots using ‘ggplot2’ library. Hierarchical clustering of samples was performed based on distance expressed as an inverse of Pearsons’s correlation coefficient and applying Ward D2 linkage algorithm. Normalized and rlog transformed expression values were scaled and centered to be relatively represented as z-scores. Heatmaps were visualized with ‘heatmap.2’ function from ‘gplots’ R library. For k-means clustering we have identified an optimal number of samples clusters with Silhouette (Rousseeuw, 1987), Elbow method (Halkidi et al., 2001), and Hubert statistics (Dalton et al., 2009), and applied a cluster number shown by minimum two of three used models. K-means clustering was conducted using ‘k-means’ function from ‘clusters’ R package with 1000 initial resampling and 20 iterations. Each gene scores were calculated as correlations with the cluster cores. Expression profiles were visualized with ‘ggplot2’ library. To identify overrepresented biological processes, gene annotation and Gene Ontology (GO) enrichment analysis were carried out using ‘clusterProfiler’ R package and hypergeometric test under α = 0.05 after *P*-value correction for multiple comparisons using FDR. A set of all genes detected under investigated conditions in all of the samples was used as a background for over-representation analyses. Gene Ontology terms were recovered from the gff3 file deposited in the IPK database with Morex V2 reference genome assembly. Over-representation results were visualized on dot-plots using internal plotting function from ‘clusterProfiler.’

#### RT-qPCR Analysis of Gene Expression

The RNAqueous Kit (Thermo Fisher Scientific) was used for RNA isolation from root meristems for RT-qPCR analysis. Root meristems were isolated in the same way and at the same time points of experiments as for RNA-seq analysis. Isolated samples were evaluated using ND-1000 spectrophotometer (Thermo Fisher Scientific). Five hundred ng of total RNA was taken for RQ1 DNase (Promega) treatment and reverse transcribed using a RevertAid First Strand cDNA Synthesis Kit (Thermo Fisher Scientific) with Oligo(dT) primers in a 20 μL reaction mix. The RT-qPCR reaction was prepared in a 10 μL volume using a LightCycler ^®^ 480 SYBR Green I Master (Roche) in two technical replicates. A volume of 2.5 μL of obtained cDNA diluted beforehand fivefold was added to the reaction mix. The primers used in the analysis were designed with Primer3 (Untergasser et al., 2012) and are listed in Supplementary Material 1. The RT-qPCR analysis was performed using a LightCycler 480 (Roche) under the following reaction conditions: initial denaturation 5 min at 95°C, followed by 10 s at 95°C, 20 s at a temperature specific for the primers, 10 s at 72°C, repeated in 40 cycles. Denaturation for the melt curve analysis was conducted for 5 s at 95°C, followed by 1 min at 65°C and heating up to 98°C (0.1°C/s for the fluorescence measurement). The qPCR efficiency and the Ct values were determined using LinRegPCR (Ruijter et al., 2009) and used for calculation of relative expression level. Two genes, *H2A* (*Histone H2A*) and *EF1* (*Translation Elongation Factor 1-a*) used as internal controls were selected based on the stability of their expression using NormFinder (Andersen et al., 2006) and BestKeeper (Pfaffl et al., 2004). The relative expression level was calculated using the ΔΔCt method (Livak and Schmittgen, 2001) and calibrated to root meristems sampled from pH = 6.0 or pH = 4.0. The *t*-Student test was applied to determine the significant differences (at *P* < 0.05) between the compared samples.

## Results

### Changes in the Barley Root System in Response to Low pH and Al Stress

**FIGURE 2:**
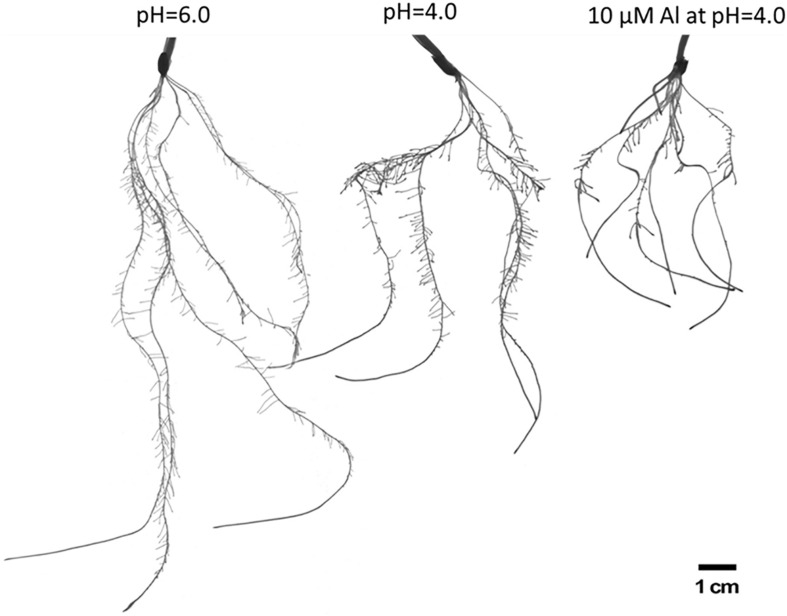
The comparison of root systems of barley cv. ‘Sebastian’ after 7 days growth in hydroponics in Magnavaca medium at pH = 6.0, pH = 4.0, and with 10 μM of bioavailable Al^3+^ ions at pH = 4.0.

To evaluate the influence of low pH and aluminum on barley root growth, we have examined in detail the root system of seedlings grown for 7 days in hydroponic conditions at pH = 6.0, pH = 4.0, and pH = 4.0 with 10 μM of bioavailable Al^3+^ ions. It has been clearly shown that low pH alone causes a significant reduction of root growth, and the addition of aluminum further inhibits its development (Figure 2).

**FIGURE 3:**
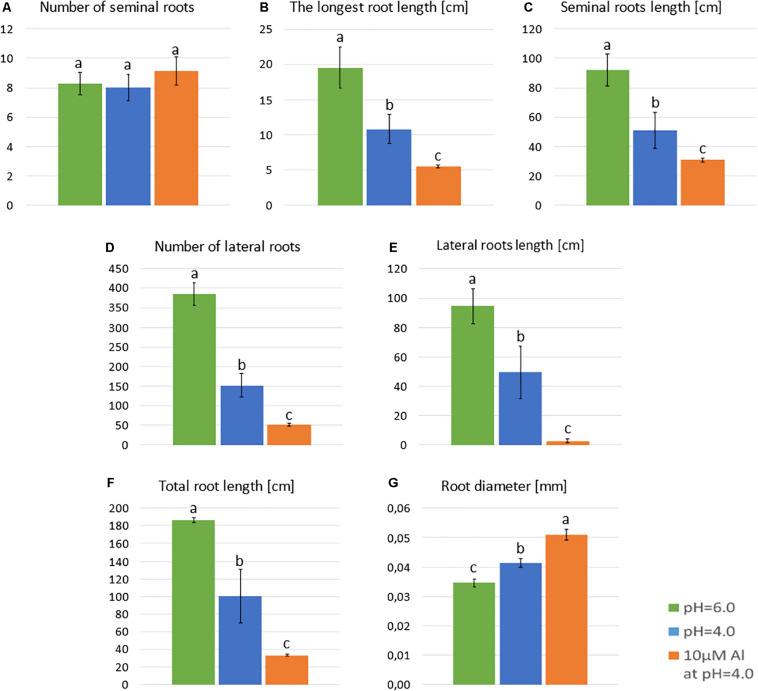
Main parameters of ‘Sebastian’ roots after 7 days growth in hydroponics in Magnavaca medium at pH = 6.0, pH = 4.0, and with 10 μM of bioavailable Al^3+^ ions at pH = 4.0. **(A)** The number of seminal roots per plant. **(B)** The length of the longest seminal root. **(C)** Total length of seminal roots. **(D)** The number of lateral roots per plant. **(E)** The length of all lateral roots. **(F)** The summary length of the whole root system (seminals + laterals). **(G)** The average diameter of roots. Statistical analyses were performed using ANOVA (*P* < 0.05) followed by Tukey’s honestly significant difference test (Tukey HSD test, *P* < 0.05). Significant differences are indicated by different letters.

Neither the low pH nor the aluminum caused any change in the number of seminal roots (Figure 3A). However, the length of the seminal roots was significantly affected by both stressors. The longest root of plants grown at pH = 4.0 were half shorter than those grown at pH = 6.0, and the longest root of plants grown in a medium with 10 μM of bioavailable Al were half shorter than those grown at pH = 4.0 (Figure 3B). Similarly, the total length of all seminal roots was reduced almost by 50% by low pH and further reduced by Al (Figure 3C). The development and growth of lateral roots of barley seedlings were affected even more. The number of lateral roots produced by the plant decreased from 385 to 152 due to the low pH (60% reduction), and to 52 due to Al exposure (65% reduction in relation to pH = 4.0) (Figure 3D). These roots were also drastically shortened. The summary length of all lateral roots was reduced by half by low pH and further reduced by 95% under aluminum stress compared to low pH conditions (Figure 3E). As a consequence, the total length of the whole root system was reduced to 53% by low pH itself and to 17% by Al stress at low pH, compared to optimal conditions of pH = 6.0 (Figure 3F). Interestingly, the diameter of the roots was also altered. Both factors, low pH and Al, caused a slight increase of root diameter (Figure 3G).

### RNAseq Data Processing Statistics

Two independent experiments, short- and long-term, were performed for transcriptome analysis with RNAseq. Nine samples were collected in the short-term experiment: three samples from root meristems grown at pH = 6.0, three samples from root meristems grown at pH = 4.0 and three samples from root meristems treated with Al for 24 h at pH = 4.0. Similarly, another nine samples were collected in the long-term experiment: from root meristems of plants grown at pH = 6.0 (three samples), pH = 4.0 (three samples) and plants treated for 7 days with Al (three samples). In total, RNA-Seq libraries were constructed from 18 samples and subjected to sequencing in the paired-end mode (PE).

**FIGURE 4:**
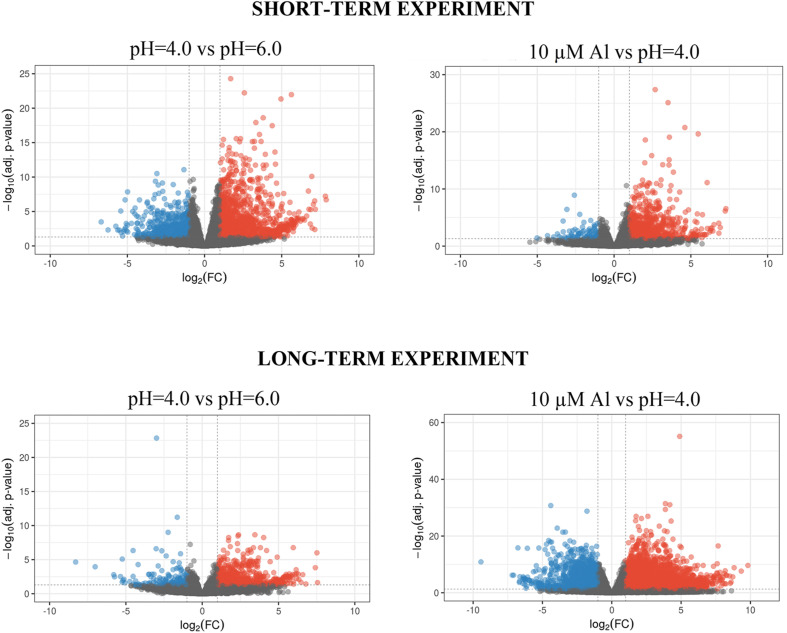
Volcano plots of differential expression analysis of barley root meristems grown at pH = 4.0 compared to pH = 6.0 and treated with 10 μM of bioavailable Al^3+^ compared to pH = 4.0 in the short-term and long-term experiments. Each dot represents one gene. Dashed lines determine cut-off of | log2FC| ≥ 1 and FDR adjusted *P*-value ≤ 0.05. Blue dots correspond to gene expression down-regulation, whereas red dots – up-regulation.

In the short-term experiment, soft trimming, filtering and exclusion of reads originating from rRNAs (main source of discarded reads) yielded a final mean per sample value of 19.2 (±3.8) mln paired end (PE) reads. On average 95.6% (±0.3%) of them were uniquely mapped to the reference genome, which indicates a high mapping rate (Table 1). In the long-term experiment, an average of 13.8 (±3.3) million clean PE reads was obtained, and a high rate of 88.4% (±4.3%) of them uniquely mapped to the barley genome (Table 1). PCA of obtained RNA-Seq data showed the significant differentiation of samples grown at pH = 6.0, pH = 4.0, and treated with Al in both experiments (Supplementary Material 2). Biological replicates from the same time-point clustered together, and PC1 explaining most of the variability (70.2% in the short-term experiment and 88.1% in the long-term experiment) corresponded to the applied treatment. The differences in gene expression were analyzed with DESeq2 tool and DEGs were identified under α = 0.05 after *P*-value FDR correction. We further analyzed genes with log_2_FoldChange (log_2_FC) ≥ 1 or ≤–1 as DEGs. In the short-term experiment, 1899 genes were differentially expressed in root meristems grown at low pH (4.0) when compared to those grown at pH = 6.0 and 986 genes were differentially expressed after exposure to Al for 24 h. In the long-term experiment, 870 genes were differentially expressed by low pH and 5873 by Al treatment for 7 days. The statistical significance of the results and magnitude of changes are shown on Volcano plots (Figure 4). To confirm obtained RNA-Seq results, four differentially expressed genes (DEGs) were checked using RT-qPCR method. The results confirmed the direction of change of expression as detected by RNA-seq (Supplementary Material 3).

**TABLE 1:** The statistics of data filtering and mapping steps for 18 analyzed PE RNA-Seq samples.

Short-term experiment				Long-term experiment			
Sample	Filtered reads	Mapped reads	Mapping rate [%]	Sample	Filtered reads	Mapped reads	Mapping rate [%]
pH6_1	22747533	21674211	95,28159	pH6_1	18337028	17344706	94,58843
pH6_2	25895857	24867094	96,02731	pH6_2	11004027	10292225	93,53144
pH6_3	16383269	15661893	95,59687	pH6_3	11440974	9777453	85,45997
pH4_1	18405817	17654616	95,91868	pH4_1	15675008	14481444	92,38556
pH4_2	13897267	13300449	95,7055	pH4_2	19387421	16840937	86,86528
pH4_3	20728621	19856210	95,79127	pH4_3	9900989	8133049	82,1438
Al_1	21361770	20383233	95,41921	Al_1	13081680	11328414	86,59755
Al_2	18438423	17588979	95,39308	Al_2	13855686	11808184	85,22266
Al_3	15398531	14667448	95,25225	Al_3	11499869	10276899	89,36536
**Average**	**19250788**	**18406015**	**95,59842**	**Average**	**13798078**	**12253701**	**88,46223**

### Global Transcriptome Analysis of Barley Root Meristems in the Short-Term Experiment

**FIGURE 5:**
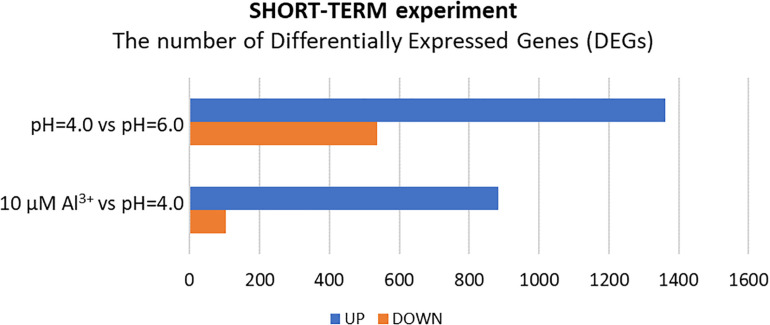
The number of differentially expressed genes (DEGs) in the transcriptome of barley root meristems in the short-term experiment. DEGs were determined for plants grown at pH = 4.0 compared to pH = 6.0; and after 24 h treatment with 10 μM Al at pH = 4.0 compared to pH = 4.0 without Al^3+^ ions (DEGs were identified under α = 0.05 with FDR adjusted *P*-value and log_2_FC ≥ 1 or ≤–1).

Surprisingly, in the short-term experiment, more genes were differentially expressed (log_2_FC ≥ 1 or ≤–1) in root meristems of barley plants grown at pH = 4.0 in relation to pH = 6.0, than in plants treated for 24 h with Al compared to plants grown at pH = 4.0 without Al (Figure 5). In total, the expression of 1899 genes was altered after 48 h of growth at low pH. Among them, 1361 were upregulated and 538 were downregulated. Treatment with 10 μM Al at pH = 4.0 for 24 h led to a change of expression of 986 genes. Majority of these genes (883) were upregulated, whereas 103 were downregulated. These numbers suggest that growing of barley seedlings in a short-term hydroponics at the low pH (4.0) has a great impact on the transcriptome profile of root meristems, even greater than short term (24 h) Al exposure at pH = 4.0 when compared to low pH conditions without Al. Correspondingly, the length of seminal roots in the short-term experiment was more affected by low pH itself than by addition of aluminum for 24 h (Supplementary Material 4).

#### Genes With Expression Altered by Low pH in the Short-Term Experiment

**FIGURE 6:**
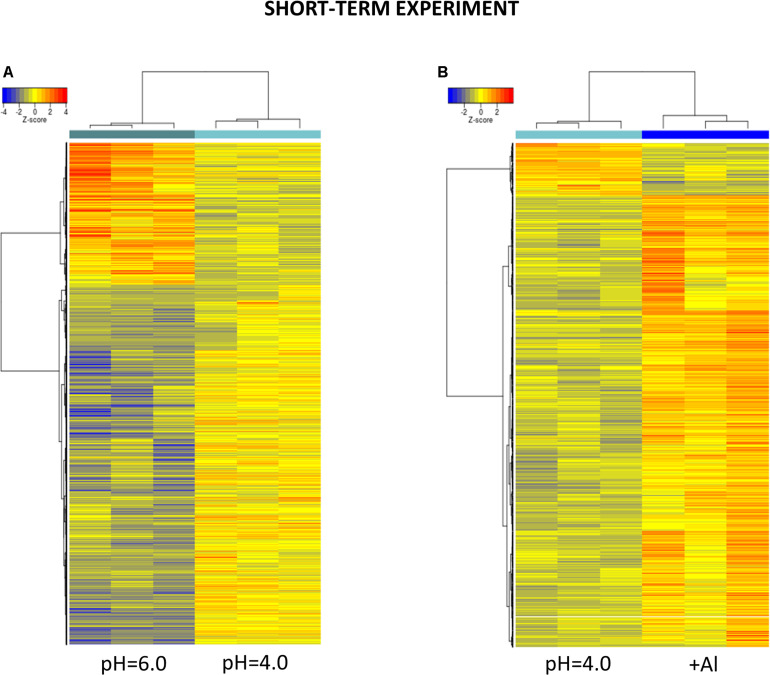
Hierarchical clustering of samples using distance based on Pearson’s correlation coefficient with Ward D2 linkage algorithm for a set of genes differentially expressed in barley root meristems in the short-term experiment. Gene counts were regularized log (rlog) transformed with library size-wise normalization, scaled and centered to be represented as *z*-scores in the log2 scale. The heat map represents the relative expression levels of DEGs. **(A)** The comparison between pH = 4.0 and pH = 6.0. **(B)** The comparison between Al-treatment at pH = 4.0 and pH = 4.0 without Al.

**FIGURE 7:**
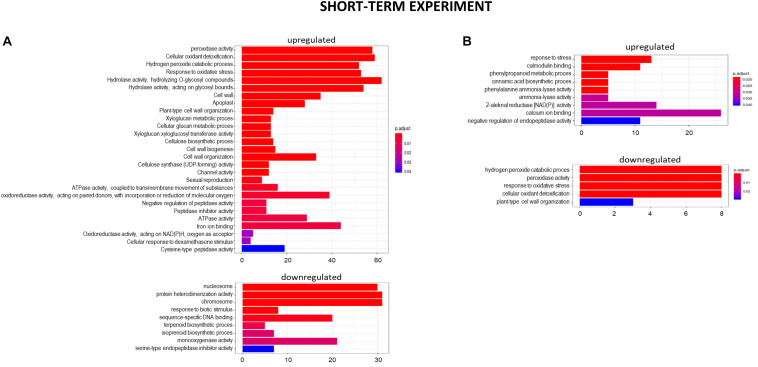
GO term enrichment of genes upregulated and downregulated in the short-term experiment by low pH **(A)** and Al **(B)**. The numbers on the *X* axis indicates the number of genes; p.adjust means FDR adjusted *p*-value.

After 48 h of seedling growth in hydroponics at pH = 4.0, 72% of DEGs were upregulated and 38% were downregulated compared to seedlings grown at pH = 6.0 (Figures 5, 6A). The GOs term enrichment allowed identification of overrepresented groups of up- and downregulated genes (Figure 7A). Among upregulated ones, a cluster of genes related to maintaining REDOX homeostasis stood out the most. The peroxidase HORVU.MOREX.r2.2HG0129730 had the highest fold change in the gene expression level (log_2_FoldChange = 7.88, Supplementary Material 5). Almost sixty other genes encoding proteins of peroxidase activity were highly upregulated by low pH. The great number of genes with the function assigned in cellular oxidant detoxification, hydrogen peroxide catabolic process or response to oxidative stress also showed increased expression. Among them are genes encoding, e.g., cytochrome P450 proteins that are monooxygenases involved in the formation of ROS (32 genes upregulated, with the highest log_2_FC = 5.68); laccases that are multicopper oxidases (12 genes upregulated, with the highest log_2_FC = 7.17); glutathione S-transferases (GSTs) that are detoxifying enzymes helping to protect cells from oxidative damage (10 genes upregulated, with the highest log_2_FC = 5.15); or aldehyde oxidases (3 genes upregulated, with the highest log_2_FC = 7.17). On the other hand, some other genes encoding proteins maintaining ROS levels were downregulated. The expression of 10 genes for cytochrome P450 (out of 16 downregulated) was highly decreased, with log_2_FoldChange > –3.0, three genes encoding GSTs and one gene encoding laccase were also downregulated, which further indicates that acidification of the environment contributes to ROS balance disruption. These data clearly show that lowering the pH from 6.0 to 4.0 induces oxidative stress in barley root meristems.

Another cluster of significantly overrepresented groups of genes upregulated at pH = 4.0 was related to cell wall development. The GOs term enrichment indicated that there are groups of genes involved, for instance, in cell wall biogenesis, cellular glucan metabolic process, xyloglucan metabolic process, xyloglucan:xyloglucosyl transferase activity, cellulose biosynthetic process, or cellulose synthase (UDP-forming) activity. Within this group, the highest change in the gene expression showed xyloglucan endotransglucosylase/hydrolase (XTH) HORVU.MOREX.r2. 4HG0348650 (log_2_FoldChange = 7.83). Fifteen genes encoding different further XTHs were identified as upregulated by low pH. These enzymes are known to cut and rejoin hemicellulose chains in the cell wall. More than 20 genes encoding expansins that are engaged in modifying the elasticity of the cell wall, and over a dozen genes encoding cellulose synthases showed increased expression pattern at low pH. Such a huge amount of DEGs related to cell wall organization evidently indicates that maintaining optimal pH is crucial for the proper development of this structure in barley roots.

Moreover, low pH influenced the signaling pathways in barley root meristems by alteration of expression of genes encoding protein kinases and transcription factors (TFs). TFs with changed expression belong mainly to WRKY (4 upregulated, 17 downregulated), MYB (7 upregulated, 6 downregulated), bHLH (7 upregulated), and NAC (5 upregulated, 2 downregulated) TFs families. Interestingly, in response to low pH, a group of genes related to chromatin organization was significantly downregulated, as for example genes encoding basic histones (H2A, H2B, and H4) or enzymes that posttranslationally modify histones, like histone-lysine *N*-methyltransferases.

The full lists of genes upregulated and downregulated by low pH (4.0) in the short-term experiment, with log_2_FoldChange ≥ 1 or ≤–1, are provided as Supplementary Materials 5, 6.

#### Genes With Expression Altered by Al Treatment in the Short-Term Experiment

After 24 h of growth in hydroponics with 10 μM of bioavailable Al^3+^ ions at pH = 4.0, almost 90% of DEGs were upregulated and only 10% were downregulated compared to pH = 4.0 without Al (Figures 5, 6B). Interestingly, the number of genes with expression affected by Al in the presented short-term experiment was twice lower than the number of genes with expression altered by low pH alone.

The GOs term enrichment identified the overrepresented groups of up- and downregulated genes after 24 h Al treatment (Figure 7B). Among them those related to the stress response were overrepresented in both, up- and downregulated groups. Out of DEGs encoding enzymes of peroxidase activity, 13 were upregulated and 7 were downregulated by 24 h of Al treatment. Four genes for glutathione S-transferases, the detoxifying enzymes, were highly upregulated. Thirteen and seven genes for cytochrome P450 were up- and downregulated, respectively. As was indicated earlier, low pH alone is already a stress factor to barley roots and this data suggest that 24 h of Al treatment additionally increases the stress.

The next overrepresented groups of upregulated genes were related to calcium homeostasis. It is well known that Al disturbs homeostasis of Ca^2+^ ions. Here, genes related to Ca^2+^ ion binding and calmodulin binding were upregulated. Calmodulin (calcium-modulated protein), activated by Ca^2+^, modifies downstream proteins such as kinases and phosphatases in the calcium signal transduction pathway.

The expression of many transcription factors was also altered (mainly upregulated) by 24 h Al treatment. The most abundant were WRKY (12 upregulated), NAC (10 upregulated), and MYB (7 upregulated) TFs. Therefore they are assumed to play important roles in regulating the expression of downstream genes involved in Al response.

The full lists of genes upregulated and downregulated by Al in the short-term experiment, with log_2_FoldChange ≥ 1 or ≤–1, are provided as Supplementary Materials 7, 8.

### Global Transcriptome Analysis of Root Meristems in the Long-Term Experiment

**FIGURE 8:**
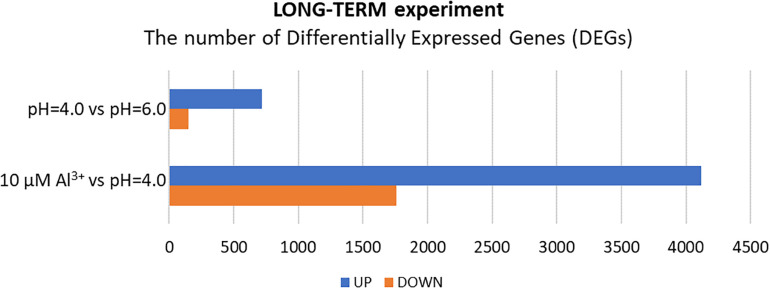
The number of differentially expressed genes (DEGs) in the transcriptome of barley root meristems in the long-term experiment. DEGs were determined for plants grown at pH = 4.0 compared to pH = 6.0; and after treatment with 10 μM Al at pH = 4.0 compared to pH = 4.0 without Al^3+^ ions (DEGs were identified under α = 0.05 with FDR adjusted *P*-value and log_2_FC ≥ 1 or ≤–1).

In the long-term experiment, the material for RNA isolation was collected 7 days after Al addition to the hydroponic solution. The seedlings were grown under conditions of optimal pH (6.0), low pH (4.0) without Al, and low pH (4.0) with 10 μM of bioavailable Al. Contrary to the results obtained for the short-term experiment, in the long-term experiment more genes were differentially expressed in root meristems of barley plants exposed to Al^3+^ ions than in plants stressed with low pH alone (Figure 8). In total, the expression of 870 genes was altered by low pH. Among them, 720 were upregulated and 150 were downregulated. Seven day treatment with 10 μM Al at pH = 4.0 led to a change of expression of a huge number of 5873 genes, of which 4116 were upregulated, whereas 1757 were downregulated. These numbers indicate that barley plants seem to adapt to low pH, at least at the transcriptome level, while the prolonged exposure to Al causes massive changes of transcriptome profile.

#### Genes With Expression Altered by Low pH in the Long-Term Experiment

**FIGURE 9:**
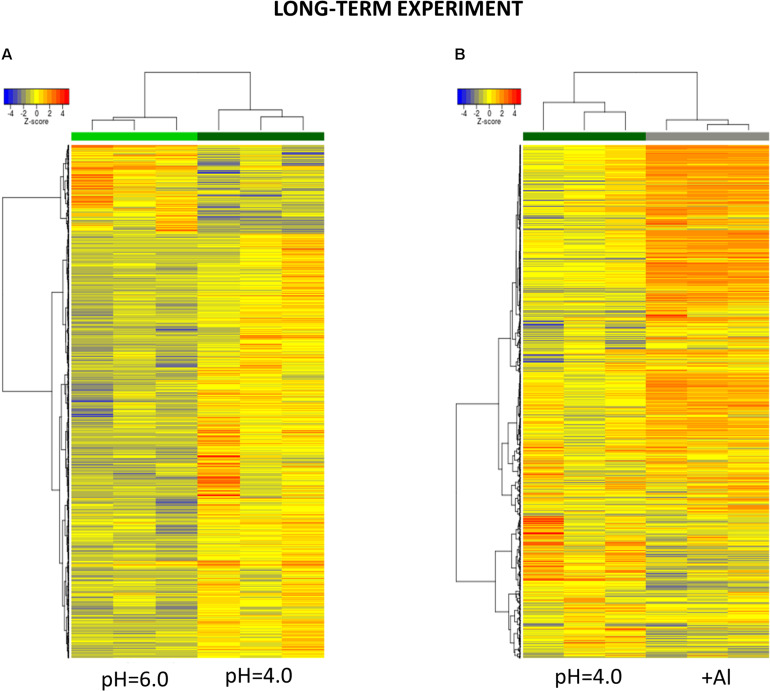
Hierarchical clustering of samples using distance based on Pearson’s correlation coefficient with Ward D2 linkage algorithm for a set of genes differentially expressed in barley root meristems in the long-term experiment. Gene counts were regularized log (rlog) transformed with library size-wise normalization, scaled and centered to be represented as *z*-scores in the log2 scale. The heat map represents the relative expression levels of DEGs. **(A)** The comparison between pH = 4.0 and pH = 6.0. **(B)** The comparison between Al-treatment at pH = 4.0 and pH = 4.0 without Al.

**FIGURE 10:**
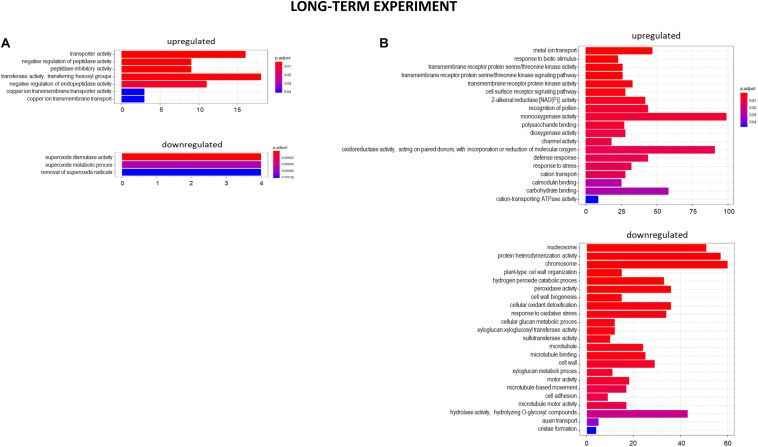
GO term enrichment of genes upregulated and downregulated in the long-term experiment by low pH **(A)** and Al **(B)**. The numbers on the *X* axis indicates the number of genes; p.adjust means FDR adjusted *p*-value.

At the 7 days time point of hydroponics at pH = 4.0, 82% of DEGs were upregulated and 18% were downregulated in relation to pH = 6.0 (Figures 8, 9A). The GOs term enrichment allowed identification of overrepresented groups of up- and downregulated genes in plants exposed to low pH (Figure 10A). The results show that among upregulated genes were those related to transporter activity, such as e.g., HORVU.MOREX.r2.3HG0242890 gene that encodes a copper transporter whose expression was highly induced by low pH (log_2_FC = 7.2). Few other genes related to copper ion maintenance were also upregulated in these conditions (CuSO_4_ is one of the components of Magnavaca solution). Another overrepresented group of upregulated genes was related to transferase activity and inhibitory regulation of peptidase activity.

The expression of genes encoding various transcription factors was also changed by low pH in the long-term experiment, however, their number was not as high as in the short-term experiment. They encoded TFs derived from the same TF families with most abundant those belonging to NAC family – eight upregulated TFs. The lower number of TFs with altered expression translated to the lower, than in the short-term experiment, number of total DEGs after exposure to pH = 4.0.

It is worth highlighting that in the short-term experiment many genes related to oxidative stress response were highly upregulated by low pH, whereas in the long-term experiment, these groups of genes were not overrepresented. For example, in total there were only 11 genes encoding enzymes of peroxidase activity upregulated after long-term exposure to pH = 4.0, in comparison to over 60 peroxidase genes upregulated by low pH in the short-term experiment. This also applies to genes encoding cytochrome P450, with over 30 of them upregulated after 48 h of hydroponics at pH = 4.0, whereas after long exposure to low pH this number dropped to 6. These findings suggest that at low pH plants are exposed to a huge oxidative stress and they need time to adapt to it.

The full lists of genes upregulated and downregulated by low pH in the long-term experiment, with log_2_FoldChange ≥ 1 or ≤–1, are provided as Supplementary Materials 9, 10.

#### Genes With Expression Altered by Al in the Long-Term Experiment

The extremely high number of genes had altered expression after 7 days of growth in hydroponics with 10 μM of bioavailable Al^3+^ ions at pH = 4.0. The majority (70%) of DEGs were upregulated and 30% were downregulated in relation to pH = 4.0 without Al (Figures 8, 9B). In the long-term experiment, the expression of significantly more genes was affected by Al than by low pH itself.

The GOs term enrichment allowed identification of overrepresented groups of genes up- and downregulated after long-term treatment with Al (Figure 10B). Based on GO term enrichment, Al seemingly causes strong oxidative stress to barley roots. Hundreds of genes involved in oxidation processes were up- and downregulated. Among them were genes with ontologies defined as e.g., monooxygenase activity, dioxygenase activity, oxidoreductase activity, acting on paired donors, with incorporation or reduction of molecular oxygen, peroxidase activity, cellular oxidant detoxification, or response to oxidative stress. Out of genes encoding peroxidases, 37 were upregulated, whereas 39 were downregulated. 72 genes encoding different proteins belonging to cytochrome P450 were upregulated and 16 were downregulated. The most upregulated gene HORVU.MOREX.r2.1HG0002460 (log_2_FC = 9.8) encodes a glutathione S-transferase and the most downregulated gene HORVU.MOREX.r2.2HG0099410 (log_2_FC = –9.4) encodes a peroxidase. Taken together, it shows that oxidative balance was disturbed in barley root meristem cells after prolonged aluminum treatment in hydroponics.

One of the overrepresented groups of genes that were upregulated in the long-term experiment was related to metal ion transport (GO:0030001). Within this GO term there are genes that may be potentially involved in the transport of any metal ion with an electric charge (therefore potentially also Al^3+^ ions) within a cell or between the cells. Three metal transporters from NRAMP (Natural resistance-associated macrophage protein) family were identified. Other metal transporters identified as differentially expressed after Al treatment were potassium, zinc, copper, magnesium, or calcium transporters. The elevated expression of genes involved in calmodulin binding further confirmed the disturbance in calcium homeostasis. Additionally, the two largest groups of upregulated transporters were heavy metal transport/detoxification superfamily (>30 upregulated, the highest log_2_FC = 5.09) and ABC transporter family proteins (>30 upregulated, the highest log_2_FC = 7.41). They might be potentially involved in Al ion transport. The downregulation of 12 genes encoding other proteins belonging to the heavy metal transport/detoxification superfamily further indicates that metal homeostasis was disturbed by exposure of roots to Al.

Upon prolonged Al treatment, genes encoding malate and citrate synthases (HORVU.MOREX.r2.2HG0146360 and HORVU.MOREX.r2.7HG0610760) were highly upregulated (with log_2_FC = 4,2 and log_2_FC = 5.5, respectively), which suggests that barley produces organic acids (OAs) in response to Al, probably to chelate Al ions in the process of detoxification. However, the gene encoding aluminum activated citrate transporter, which is a membrane protein involved in the exudation of citrate outside the root cells, was not upregulated, and aluminum activated malate transporter was even downregulated (log_2_FC = –1.83).

Among genes downregulated by Al were those related to chromosome organization, e.g., genes encoding basic histones (H2A, H2B, H3, and H4) and enzymes that modify histones, e.g., histone deacetylase 2 or histone *N*-methyltransferases. The other overrepresented group of downregulated genes was related to the cell wall development, which is consistent with the assumption that Al inhibits cell wall growth. What is more, many genes that were downregulated upon Al treatment for 7 days were involved in microtubule binding, movement, and activity. Al binds to the cytoskeleton and disrupts spatial orientation of the cytoskeleton, which disturbs cell expansion and is consistent with our observation that genes involved in microtubule organization are also Al-responsive. Taken together, the downregulation of the mentioned genes clearly indicates that the growth of cells in root meristem is disturbed and slowed down.

Genes encoding kinases were highly overrepresented within upregulated genes, which indicates activation of signaling pathways. The expression of many various transcription factors was also strongly altered by 7 days Al treatment which further resulted in the extremely high number of DEGs. Significantly more TFs were upregulated than downregulated and among them were those belonging to e.g., MYB (41 upregulated, 8 downregulated), WRKY (35 upregulated, 2 downregulated), NAC (26 upregulated, 1 downregulated), and bZIP (12 upregulated, 3 downregulated) TF families.

The full lists of genes upregulated and downregulated by Al treatment in the long-term experiment, with log_2_FoldChange ≥ 1 or ≤–1, are provided as Supplementary Materials 11, 12.

### Common Genes With Expression Altered by Low pH and Al Treatment

**FIGURE 11:**
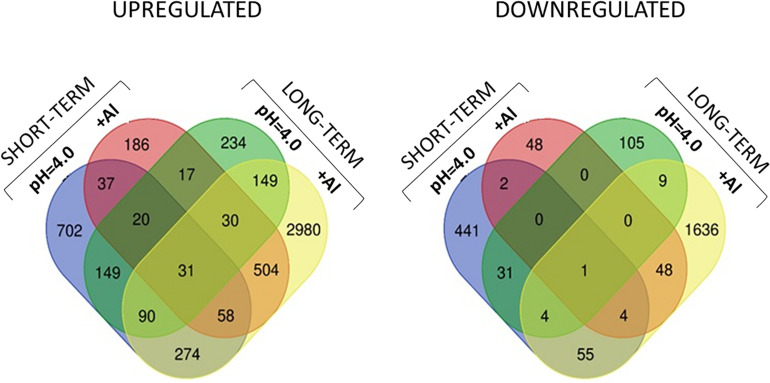
Venn diagrams showing common and specific DEGs upregulated and downregulated between all experimental combinations.

**FIGURE 12:**
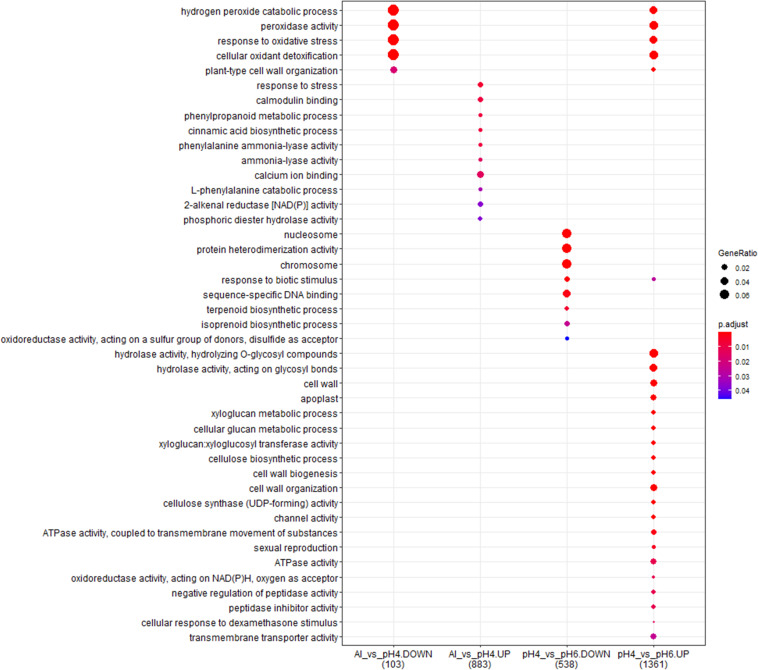
The comparison of GO terms over-representation analysis of differentially expressed genes in the short-term experiment using hypergeometric test under α = 0.05 with FDR adjusted *P*-value.

**FIGURE 13:**
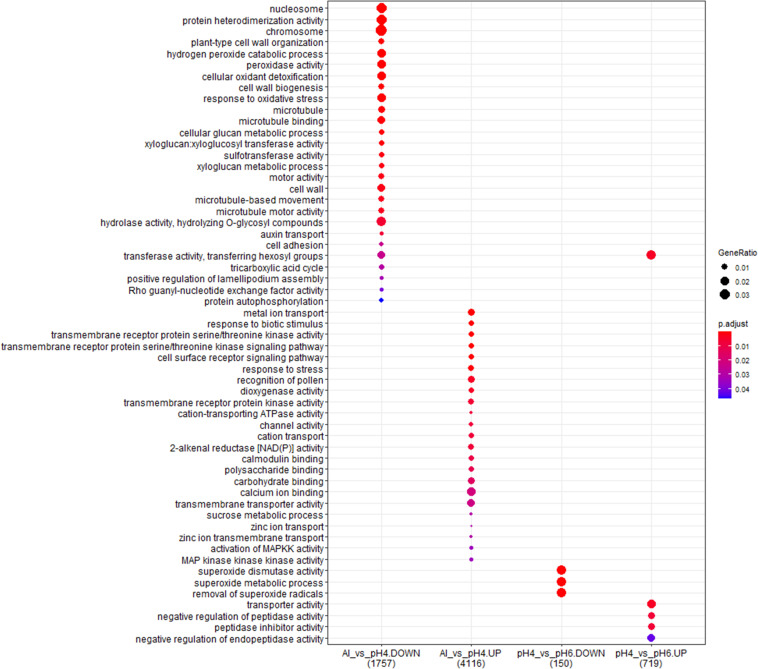
The comparison of GO terms over-representation analysis of differentially expressed genes in the long-term experiment using hypergeometric test under α = 0.05 with FDR adjusted *P*-value.

The comparison of DEGs between transcriptomes of low pH- and Al-treated barley root meristems showed that only a small group of DEGs was shared and the expression of much more genes was changed specifically by each treatment (Figure 11). It indicates the activation of distinct molecular mechanisms in response to these stresses. Moreover, the comparative analysis of GO terms enrichment further indicated that low pH and Al stress altered the expression of different groups of genes with diverse molecular functions, both in the short- and long-term experiments (Figures 12, 13).

**FIGURE 14:**
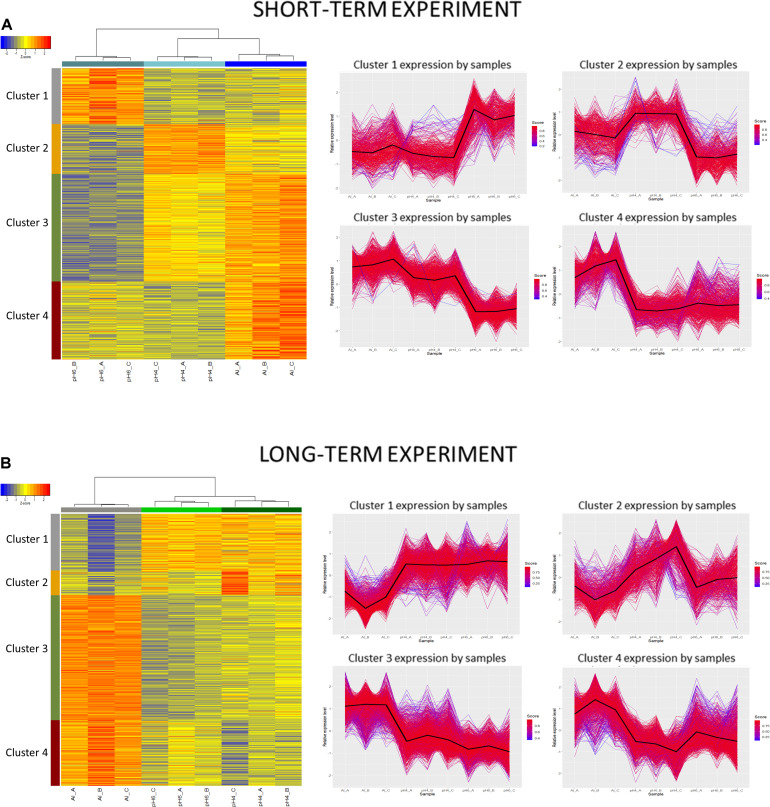
Clustering analysis using hierarchical clustering of samples and k-means clustering of genes. Clustering was performed using a selected set of genes differentially expressed in barley root meristems grown at pH = 4.0 compared to pH = 6.0 and treated with 10 μM of bioavailable Al^3+^ compared to pH = 4.0. Gene counts were regularized log (rlog) transformed with library size-wise normalization, scaled and centered to be represented as *z*-scores in the log2 scale. Hierarchical clustering of samples was performed using distance based on Pearson’s correlation coefficient with Ward D2 linkage algorithm. Heat-map represent their expressional patterns. Color bars on the left are corresponding to each consequent cluster identified: from 1 on the top to the 4 at the bottom. For each gene cluster detected with k-means clustering, a plot of relative gene expression profile is shown on the right with black lines indicating each cluster centroids. Scores represent correlations of each gene with the cluster core. **(A)** Short-term experiment; **(B)** long-term experiment.

However, in the short-term experiment, there were 153 DEGs common for low pH and Al treatment (146 upregulated and 7 downregulated). Among them were some genes related to oxidative stress response (e.g., encoding peroxidases or alpha-dioxygenase 2) and cell wall development (e.g., encoding pectate lyase, pectinesterase, xyloglucan endotransglucosylase/hydrolase and expansin). Additionally, several transcription factors were also upregulated by both analyzed stresses (low pH and Al), which indicates that some common mechanisms of response might be activated. The lists of common genes with expression altered by low pH and Al in the short-term experiment is provided together with their annotations as Supplementary Material 13. Additionally, to illustrate the prevalent expression patterns of DEGs in the short-term experiment, we performed the analysis of gene expression profiles using k-means clustering. Four clusters of genes with prevailing expression patterns have been identified (Figure 14A). DEGs common for both analyzed factors, with expression upregulated by low pH and further upregulated by Al are grouped within the Cluster 3. The GO term enrichment of these group of genes also showed that they are mainly related to oxidative stress and cell wall development (Supplementary Material 14). The GO term enrichment of DEGs from remaining clusters is also provided in Supplementary Material 14.

In the long-term experiment, there were 314 DEGs common for low pH and Al treatment (300 upregulated and 14 downregulated). Similarly as in the short-term experiment, among common genes with expression altered after long low pH and Al treatments there were DEGs related to oxidative stress (e.g., encoding peroxidases or cytochrome P450 family proteins) and cell wall development (e.g., encoding xyloglucan endotransglucosylases/hydrolases or aldehyde dehydrogenase). The list of common genes with expression altered by low pH and Al in the long-term experiment is provided together with their annotations as Supplementary Material 15. This data indicate the existence of common responses to low pH and Al, nevertheless, the majority of DEGs showed expression changes specifically in response to one of these factor, with the highest number of genes with expression affected after long Al exposure. This is also illustrated by k-means clustering which showed four clusters of genes with prevalent expression patterns in the long-term experiment (Figure 14B). The presented heatmap shows that in most clusters (except for a small Cluster 2) aluminum altered the gene expression to the greatest extent. DEGs common for low pH and Al with expression upregulated by both analyzed factors independently are grouped within the Cluster 3. The overrepresented GO terms in Cluster 3 were e.g., monooxygenase activity, iron ion binding, oxidoreductase activity, transmembrane transporter activity, calcium ion binding and metal ion transport. The GO term enrichment of DEGs from all clusters is provided as Supplementary Material 16.

## Discussion

Barley (*Hordeum vulgare* L.) is the most Al-sensitive species among small grain cereals, but still there are differences in Al tolerance among barley cultivars, which are mostly correlated with the ability of the genotype to secrete citrate (Zhao et al., 2003; Furukawa et al., 2007). Cv. ‘Sebastian’ used in our study is relatively tolerant to Al when compared to other barley cultivars (Vega et al., 2019). Nonetheless, even the micromolar concentration of bioavailable Al^3+^ ions (10 μM Al^3+^) applied in hydroponic solution at pH = 4.0 for 7 days, extremely reduced (by 83%) the total length of ‘Sebastian’ roots, compared to root length of untreated plants grown at optimal pH = 6.0. However, without a doubt, the reduction of root growth was not caused by Al^3+^ ions only, but also by the low pH and proton/H^+^ toxicity, as growing plants at pH = 4.0 without addition of Al reduced the total root length of ‘Sebastian’ seedlings almost by half. It has also been previously reported that barley is very sensitive to H^+^ toxicity (Zhao et al., 2003; Guo et al., 2004). Similarly, higher H^+^ activity, significantly decreased the root length of rice seedlings grown at pH = 3.5 and pH = 4.5 compared to pH = 5.5 (Zhang et al., 2015). However, even though it was reported that proton rhizotoxicity can be more harmful than Al rhizotoxicity in natural acid soils (Kinraide, 2008), the effect of the low pH itself has been understudied in the aspect of Al toxicity. It has to be stressed that in natural conditions, in acidic arable lands, Al toxicity and H^+^ toxicity coexist and together negatively affect barley performance and yield. To discriminate Al effect from a low pH effect in our RNA-Seq analysis, we compared transcriptomes of Al treated root meristems to those grown without Al at pH = 4.0. Additionally, we also compared the transcriptomes of barley plants grown at pH = 4.0 to those grown at pH = 6.0 (both without Al).

Our results show that the low pH caused global changes in barley transcriptome profile when seedlings were grown in hydroponics for 48 h (short-term experiment), whereas after a prolonged time of growth under low pH (further 7 days), the number of DEGs significantly decreased, suggesting that partial adaptation of plants to this stress occurred. Interestingly, the opposite effect was seen in regards to aluminum toxicity. After 24 h of Al treatment, many genes were up- and downregulated in root meristems, however, their number increased extremely after 7 days of Al treatment, which suggests that remodeling of the transcriptome following Al stress is a long-lasting and dynamic process. These results are in agreement with the microarray analysis of *Arabidopsis thaliana* transcriptome profiles in response to Al stress, where more transcripts were Al-responsive after 48 h than 6 h treatment (Kumari et al., 2008).

### Low pH and Al as Oxidative Stressors

Different abiotic stresses, such as drought, cold, salt, and heat, can disrupt the balance of ROS content and lead to their accumulation in the cell, which results in oxidative stress (reviewed in You and Chan, 2015). It has long been known that aluminum also induces oxidative stress in plants. The first genes related to oxidative stress, which were identified as being upregulated by Al in *Arabidopsis thaliana*, encoded peroxidase, glutathione-S-transferase, and protein homologous to the reticuline:oxygen oxidoreductase enzyme (Richards et al., 1998). Al was found to influence reactive oxygen intermediates, lipid peroxidation, protein oxidation, and activities of antioxidant enzymes in many different plant species, including *Allium cepa* (Achary et al., 2008), *Triticum aestivum* (Xu et al., 2012; Sun et al., 2017), and *Zea mays* (Boscolo et al., 2003; Giannakoula et al., 2010). In the presented study, we show that both analyzed factors, low pH and Al, led to the alteration of oxidative stress genes expression in barley roots.

In our ‘low pH only’ study, the number of DEGs related to oxidative stress response was very high after 48 h of growth at pH = 4.0, but in the long-term experiment, the number of DEGs significantly decreased, suggesting that barley plants adapt to oxidative stress caused by low pH (H^+^ toxicity) over time. The study performed in rice (*Oryza sativa*) has shown that growing plants for 2 weeks at pH lowered to 3.5 led to the serious lipid peroxidation and increases of H_2_O_2_ and MDA (malondialdehyde) content in rice roots. At the transcriptomic level, it led to downregulation of copper/zinc superoxide dismutases (*Cu/Zn SOD1*, *Cu/Zn SOD2*) and catalases (*CATA* and *CATB*), and upregulation of ascorbate peroxidase 1 (*APX1*). Correspondingly, the activity of these enzymes was also altered. It was assumed that higher activity of APX can contribute to adaptation of rice to low pH (Zhang et al., 2015). In our study, the expression of genes encoding SODs was not altered after 48 h of growth at low pH, but the extension of hydroponic culture to 7 days caused a significant decrease in expression level of four of Cu/Zn SODs, similarly to rice. However, the genes encoding ascorbate peroxidase or CATA were not found among DEGs and the barley ortholog of *CATB* was even highly upregulated (log_2_FC = 4.56), which may be related to the higher sensitivity of barley to low pH compared to rice.

In the case of Al treatment, a high number of genes related to oxidative stress were differentially expressed in barley roots, especially in the long-term experiment, where hundreds of these genes were highly up- and downregulated. Among them there were genes encoding peroxidases (PODs), superoxide dismutases (SODs), cytochrome P450 monooxygenases, glutathione S-transferases (GSTs), thioredoxins (TRX), and others. Studies performed on other species, e.g., Arabidopsis, cucumber (*Cucumis sativus*), rice, wheat (*Triticum aestivum*) and citrus (*Citrus sinensis* and *Citrus grandis*) also showed that Al induces strong oxidative stress and upregulates the activity of antioxidative mechanisms (Kumari et al., 2008; Pereira et al., 2010; Ma et al., 2012; Guo et al., 2017; Liu et al., 2018; Awasthi et al., 2019). In general, when different genotypes were compared after Al treatment, the Al-tolerant lines were characterized by a higher activity of the antioxidative system than the Al-sensitive ones. However, RNA-seq analysis in maize showed that the total number of genes related to oxidative stress upregulated by Al treatment was higher in the Al-sensitive than the Al-tolerant genotype, suggesting that upregulation of these genes was merely a consequence of Al toxicity, not the activation of Al tolerance mechanisms (Maron et al., 2008). Such a huge number of DEGs from this category in our experiment emphasizes the very high level of Al-sensitivity of barley compared to other species, even though ‘Sebastian’ cultivar belongs to the Al-tolerant group among barley cultivars.

Peroxidases are known to be enzymatic antioxidants, hence massive upregulation in their expression means that the plant is under oxidative stress. In the presented study, 61 genes encoding peroxidases were upregulated after 48 h growth at low pH, but after further 7 days of low pH hydroponics, this number dropped to 11 DEGs. The same period (7 days) of Al treatment caused alteration of expression of many more genes encoding different peroxidases, which were both up- (37 POD genes) and downregulated (39 POD genes). It should be noted that PODs have more diverse functions, e.g., they are involved in cross-linking of the cell wall constituents (Bakalovic et al., 2006). The oxidative cross-linking of the cell wall components managed by some classes of PODs may increase cell wall stiffening and decrease its extensibility which is associated with inhibition of root growth by Al (Ma et al., 2012). In the presented experiments, the 7-day treatment of barley seedlings with Al^3+^ ions caused a significant reduction of root growth accompanied by the increase of root diameter. Moreover, the activity of some PODs that leads to H_2_O_2_ formation may be a potential mechanism of Al tolerance, because production of H_2_O_2_ may be used to restructure the cell wall and block Al entry by decreasing cell wall porosity (Maron et al., 2008). What is more, Tamás et al. (2005) presented that the production of H_2_O_2_ mediated by PODs in response to Al led to cell death of barley root border cells and hence protected root tips by chelating Al in the dead cells. The cell wall-bound PODs were also found to be involved in lignin biosynthesis, which is known to be one of the symptoms of Al stress (Li et al., 2003). The contrasting pattern of increased and decreased expression of different peroxidase genes in response to Al treatment has been observed also in other transcriptomic studies (Kumari et al., 2008; Maron et al., 2008; Li et al., 2017). It shows the complex and diverse roles of peroxidases in Al stress response.

Another example of enzymes involved in oxidative stress response is thioredoxins (TRX) - thiol-oxidoreductases that function in maintaining redox homeostasis (Meyer et al., 2012). They are induced by a variety of oxidative stimuli and their overexpression protects the cell from cytotoxicity caused by oxidative stress (Nishinaka et al., 2001). Lately, the *AtTRX1* (*Thioredoxin H-type 1*) gene was identified in GWAS studies in Arabidopsis and confirmed by reverse-genetics and co-expression gene network analysis as associated with Al-tolerance (Nakano et al., 2020). However, to the best of our knowledge, to date there was no report about TRX involvement in Al tolerance or response in monocots. Interestingly, in our study the expression of *THR* genes was altered mainly by Al (two genes upregulated in the short-term and 11 up- and 3 down-regulated in the long-term experiment), whereas low pH affected the expression of only two of *THR* genes in both, the short- and the long-term treatment. The Arabidopsis knock-out mutant in the *TRX1* gene was hypersensitive to Al, but not to proton (low pH) toxicity (Nakano et al., 2020). These data together suggest that thioredoxins are involved in the protection of cells from Al-induced oxidative stress rather than from the proton-induced one.

### Cell Wall Related Genes Regulated by Low pH and Al

The cell wall is suggested to be a primary target of Al toxicity and the majority of Al absorbed by the root tissue is localized in the apoplast. Aluminum binds to the negatively charged carboxylic groups of pectins and changes the cell wall properties, which may cause inhibition of the root cell elongation and growth (Kochian, 1995; Zheng and Yang, 2005; Silva, 2012). Our RNA-Seq data show that the expression of several genes encoding enzymes that directly modify pectins (with GO:0042545 – cell wall modification) was affected, mainly by the prolonged Al treatment. However low pH itself also changed the expression profile of some genes from this group, although to a lesser extent, which suggests that Al stress has a larger impact on modifying pectins in barley root meristematic cells than low pH alone. It is in line with our previous study showing that aluminum changes the pectin cell wall composition in barley root cells (Jaskowiak et al., 2019). Barley plants exposed to a long Al exposure showed the changes in content and localization of the pectic epitopes involved in maintenance of cell wall flexibility, stiffening of the wall and firmness of the cells. In the presented study, among DEGs related to pectin modification, those encoding pectinesterases and pectin lyases were the most abundant, especially after long-term Al treatment. Pectinesterases (also known as pectin methylesterases) belong to a large family of isozymes that catalyze the de-esterification of pectins. In our study, the expression of several genes encoding pectinesterases was upregulated by both, low pH and Al. It is consistent with our previous studies where we show, by analyzing LM19 and LM20 antibodies, that unesterified homogalacturonans (HGs) were more abundant in the Al-treated roots compared to the not treated ones (Jaskowiak et al., 2019). Similar results were obtained previously for maize, which additionally supports the hypothesis that the difference in Al tolerance among maize genotypes may depend on the level of methyl-esterification of pectins (Eticha et al., 2005).

It has been reported that Al stress enhances the incorporation of lignin into the cell wall in roots of many plant species, including wheat and rice (Hossain et al., 2005; Sasaki et al., 2006; Wang and Kao, 2007). The deposition of lignin provides the rigidity and mechanical resistance of the plant cell wall by creating a barrier that limits the radial movement of metals and pathogens (Gavnholt and Larsen, 2002). Phenylalanine ammonia-lyase (PAL) is an enzyme involved in the biosynthesis of lignin. In our study, genes encoding PALs were upregulated specifically after Al treatment (five and seven genes upregulated in the short- and long-term experiment, respectively). As indicated earlier, some DEGs encoding peroxidases with expression altered in the presented RNA-seq data may also be related to lignin biosynthesis. Another group of enzymes that may be involved in lignin deposition are laccases, because of their localization in lignifying cell walls and their potential to oxidize lignin precursors (Gavnholt and Larsen, 2002). In our study, the genes encoding laccases were upregulated by both, low pH and Al, with the highest response after 7 days Al treatment. They were also found to be up-regulated by Al in maize (Maron et al., 2008). These data indicate that lignin deposition plays a role in plant response to Al toxicity as a potential cause of root growth inhibition. It can possibly play a role in Al tolerance by blocking the entrance of Al inside the root tissue.

### Transcription Factors Modulated by Low pH and Al

Various TFs were overrepresented among genes with expression changed by low pH and Al. They belong mainly to WRKY, MYB, and NAC families and they all may play complementary roles in regulating the expression patterns of low pH- and Al-responsive genes. The differences in TFs expression profiles between Al and low pH treatments indicate that various responses may be activated upon these two stresses.

It is well documented that one transcription factor is of special importance in both, low pH and Al tolerance in Arabidopsis – the C_2_H_2_ zinc-finger protein STOP1 (Sensitive To Proton rhizotoxicity 1). STOP1 regulates multiple genes protecting Arabidopsis from H^+^ and Al toxicities and *stop1* mutants (T-DNA insertional, as well as missense) are H^+^- and Al-hypersensitive. Their hypersensitivity is related to downregulation of *AtALMT1* (*Aluminum-Activated Malate Transporter1*), *AtALS3* (*Aluminum-Sensitive 3*) that encodes an ABC transporter possibly involved in redistribution of Al, and other genes involved in ion homeostasis and metabolic pathways regulating pH (Sawaki et al., 2009). OsART1 (Aluminum Resistance Transcription factor 1), the ortholog of AtSTOP1, activates multiple genes involved in Al tolerance in rice, including those implicated in external and internal Al detoxification, e.g., *STAR1* (*Sensitive to Al rhizotoxicity 1*) encoding ABC transporter. However, unlike STOP1 in Arabidopsis, OsART1 is involved specifically in Al response only, not in response to the stress caused by low pH (Yamaji et al., 2009). More intriguingly, the VuSTOP1 (ortholog found in rice bean, *Vigna umbellata*) is involved mainly in response to H^+^ toxicity (Fan et al., 2015). Expression of *AtSTOP1* and *OsART1* turned out to be constitutive and not affected by proton or Al stress, hence these TFs are thought to be regulated posttranslationally. It was recently confirmed that in Arabidopsis AtSTOP1 function is regulated by SUMOylation (Fang et al., 2020). On the contrary, the expression of *VuSTOP1* is induced by both, H^+^ and Al^3+^ (reviewed in Fan et al., 2016). What is more, in wheat three homoeologous *TaSTOP1* genes display differential expression patterns: *TaSTOP1-A* is induced by Al^3+^, *TaSTOP1-B* is constitutively expressed and *TaSTOP1-C* is induced by H^+^ (Garcia-Oliveira et al., 2013). We used *TaSTOP1* sequence to search for potential barley orthologs and we found one barley *STOP1* ortholog: HORVU.MOREX.r2.3HG0249360. It encodes a zinc finger protein with DNA-binding transcription factor activity. The expression pf *HvSTOP1* was not affected in the presented study neither by low pH nor by Al, however its GOs indicated that it is involved in both, low pH and Al response (GO:0010044 – response to aluminum ion, GO:0010447 – response to acidic pH). Thus, it may be assumed that barley *HvSTOP1* gene is regulated posttranslationally, similarly to the STOP1 in Arabidopsis.

### Transporters Specific for Al Response

Other interesting groups among DEGs encode different types of transporters that were differentially expressed especially in long-term experiments. Three metal transporters, specifically upregulated only by Al, encode NRAMP proteins (Natural Resistance-Associated Macrophage Protein). One of them, HORVU.MOREX.r2.7HG0610240 (log_2_FC = 1.35) is homologous to *ZmNrat1* (*nramp Aluminum Transporter 1*) that is known to be a membrane transporter of aluminum in maize (Guimaraes et al., 2014; Matonyei et al., 2020). Similar to the barley gene, *ZmNrat1* was also upregulated by Al treatment. It is suggested that NRAT1 membrane proteins are involved in the Al response mechanism by being responsible for the transport of Al from outside to inside the cell, which reduces Al concentration in the apoplast.

In our study genes encoding potassium, zinc, or copper transporters were found to be differentially expressed by both applied stresses. However, magnesium transporters were activated only by Al. The examples are genes: HORVU.MOREX. r2.3HG0249560 encoding magnesium transporter MRS2-like protein, which is an ortholog of *OsMGT1* (*Magnesium Transporter 1*), upregulated after both short and long Al treatment and HORVU.MOREX.r2.2HG0180770 encoding another putative magnesium transporter whose expression increased significantly only after long Al treatment. Similarly, which was demonstrated in rice, *OsMGT1* expression was rapidly upregulated by Al, but not by low pH and was found to be regulated by OsART1 (Chen et al., 2012). This transporter is responsible for Mg uptake in the roots and increasing internal Mg^2+^ concentration was demonstrated to be crucial for conferring Al tolerance (reviewed in Rengel et al., 2015).

ABC (ATP-Binding Cassette) transporters are a large family of ubiquitous transmembrane proteins responsible for the active transport of various ligands across membranes (reviewed in Linton, 2007). Some representatives of this group are confirmed to be involved in detoxifying Al. A great number of genes encoding ABC transporters were upregulated in our study by Al. Also many were upregulated by low pH, but only in the short-term treatment experiment. Two genes encoding ABC transporters that were found among Al specific DEGs were homologous to *OsALS1* (*Aluminum Sensitive1*, Os03g0755100), namely HORVU.MOREX.r2.5HG0424840 and HORVU.MOREX.r2.5HG0424850 (lof_2_FC = 1.4 and 3.15, respectively). *OsALS1* encodes a tonoplast-localized ABC transporter and is regulated by OsART1. Its expression in rice was also specifically induced by Al, not by low pH, as it is responsible for sequestration of Al into the vacuole (Huang et al., 2012). However, the expression of Arabidopsis ortholog, *AtALS1*, was not Al inducible (Larsen et al., 2007). Because of the increase of the expression of two barley *ALS1* orthologs in response to Al (similarly as in rice), it may be assumed that both of them are potentially involved in internal Al detoxification in barley.

OsSTAR1/STAR2 complex is another example of an ABC transporter that is responsible for Al detoxification in rice. *OsSTAR1* encodes an ATP-binding protein that forms a complex with a transmembrane protein OsSTAR2. The STAR1/STAR2 complex is responsible for the transport of UDP (uridine diphosphate)-glucose that can modify cell walls and therefore mask Al-binding sites. Both *OsSTAR1* and *OsSTAR2* are upregulated upon Al stress in rice (Huang et al., 2009). In Arabidopsis knock-out of *AtSTAR1* resulted in increased sensitivity to Al, however its expression was constitutive in roots and shoots and was not induced by Al (Huang et al., 2010). However, the expression of *AtALS3*, that is homologous to *OsSTAR2*, increased in roots following Al exposure (Larsen et al., 2005). The homologs of *OsSTAR1* and *OsSTAR2* were identified in the barley genome but only a homolog of *OsSTAR1*, HORVU.MOREX.r2.4HG0339800, was upregulated in our RNA-seq experiment after a prolonged Al treatment (log_2_FC = 1.35). These data suggest that the pathway of Al tolerance based on Al detoxification is not as efficient in barley as in rice, which is consistent with rice being a more highly Al-tolerant cereal.

### OA Related Genes

So far, the best-documented mechanisms that help higher plants to cope with Al toxicity rely on organic acid (OA) exudation, which can chelate and thus neutralize Al^3+^ ions. OAs can act either in the rhizosphere when they are released outside the root tissue (Al exclusion mechanism), or inside the cell where they take part in Al detoxification (Al tolerance mechanism). Different plant species may secrete different OAs from roots, mainly citrate, malate, and/or oxalate anions (reviewed in Yang et al., 2013, 2019). In general, species or varieties that are tolerant to Al can secrete high levels of OAs when exposed to Al stress (e.g., Li et al., 2000; Yang et al., 2000; You et al., 2005; Dong et al., 2008). In barley, the most Al-sensitive among small grain cereals, the differential Al tolerance observed among different cultivars is correlated mainly with citrate secretion (Zhao et al., 2003). Correspondingly, in our study, the prolonged Al treatment caused a very high upregulation of a gene encoding citrate synthase (HORVU.MOREX.r2.7HG0610760 with log_2_FC = 5.5), but also an increased expression of a gene encoding malate synthase (HORVU.MOREX.r2.2HG0146360 with log_2_FC = 4.2). Interestingly, low pH alone also caused upregulation of citrate synthase (with log_2_FC = 6.0), but only in the short term experiment.

Organic acids produced by plants are exuded outside the root to the rhizosphere through membrane transporters. The first OA transporter, a malate transporter ALMT1 (Aluminum-activated Malate Transporter 1) was discovered in wheat. The *TaALMT1* gene is constitutively highly expressed in the Al-tolerant wheat cultivars and its expression is not upregulated by Al (Sasaki et al., 2004). In a tea plant that is highly tolerant to Al, four genes encoding ALMT homologs were found and contrary to wheat, all of them were upregulated by Al (Li et al., 2017). In our study the *HvALMT1* gene was not upregulated and was even slightly downregulated by Al. It is in line with the fact that in response to Al, barley plants release only citrate but not malate to the rhizosphere (Zhao et al., 2003). The increase in the expression level of malate synthase after Al treatment may indicate that malate is involved in internal detoxification of Al. Nevertheless, the overexpression of the *TaALMT1* gene increased the malate secretion and Al tolerance in transgenic barley (Delhaize et al., 2004).

The transmembrane transporters releasing citrate anions outside the cells were first identified in barley and sorghum (*Sorghum bicolor*) and named, respectively: HvAACT1 (Aluminum Activated Citrate Transporter 1) (Furukawa et al., 2007) and SbMATE1 (Magalhaes et al., 2007). Afterward they were identified in many other plant species, including wheat, maize, rye, rice, and rice bean (Ryan et al., 2009; Maron et al., 2010; Yokosho et al., 2010, 2011; Yang et al., 2011). These citrate transporters belong to the MATE (Multidrug And Toxic Compound Extrusion) family that is one of the largest plant transporter families. In the majority of plant species, genes encoding these transporters are upregulated by Al. Surprisingly, *HvAACT1* was not found to be upregulated by Al stress in barley (Furukawa et al., 2007). The barley cultivars that are relatively tolerant to Al were characterized by constitutive high expression of *HvAACT1*. In our study, we also did not find *HvAACT1* among DEGs in any experimental combination, which indicates that its expression is not altered by low pH or Al.

## Conclusion

Here we show for the first time the global transcriptome analysis of root meristematic cells of barley *Hordeum vulgare* L. grown at low pH and treated with Al. We provide a full list of differentially expressed genes that may be useful for studying mechanisms of H^+^ and Al toxicity in this important crop species. The obtained results provide new insights into the very complex mechanisms underlying H^+^ and Al tolerance in barley, suggesting that there are several common, but many more specific genetic pathways launched in response to these stresses. The fact that many various mechanisms are activated indicates that the pyramiding of genes for H^+^- and Al-tolerance to obtain higher tolerance in barley is possible. Based on our results we can definitely say that both factors, low pH and Al, are the enemies of barley. However, aluminum causes more changes at transcriptome level when plants are exposed for this stress for a long time. It should be noted that plants grown on acidic soils are simultaneously exposed to low pH and Al throughout their life.

## Data Availability Statement

RNA-Sequencing data reported in this article has been deposited in the Gene Expression Omnibus under the accession no. GSE167271.

## Author Contributions

IS, MS-Z, MK, and PL conceived the study. IS, MS-Z, and MK designed the experiments. MS-Z and MNa performed the low pH and Al experiments. MNa analyzed the root parameters. MS-Z, KC, and MG isolated RNA for RNA-seq and qPCR analysis. MG performed the qPCR analysis. MNi prepared the libraries for RNA-seq and performed sequencing in the short-term experiment. KC and MK performed the bioinformatic analysis. MS-Z, IS, and PL interpreted the results. MS-Z wrote the manuscript. IS and PL revised and edited the manuscript. All authors have approved the manuscript.

## Conflict of Interest

The authors declare that the research was conducted in the absence of any commercial or financial relationships that could be construed as a potential conflict of interest. The handling editor declared a past co-authorship with the authors MS-Z and IS.

## Footnotes

1 https://smartroot.github.io/SmartRoot-Installation/

2 https://github.com/najoshi/sickle
