# Supplementary material for: Aluminum or Low pH – Which Is the Bigger Enemy of Barley? Transcriptome Analysis of Barley Root Meristem Under Al and Low pH Stress
Source: Front Genet. 2021 May 19;12:675260. doi: 10.3389/fgene.2021.675260 (PMC8244595; doi:10.3389/fgene.2021.675260)
Supplement: Supplementary file 1 [file Data_Sheet_1.zip › Table 1.DOCX]

| **Gene** | **Annotation** | **Primer Forward** | **Primer Reverse** |
| --- | --- | --- | --- |
| Reference 1 - EF1 | Translation Elongation Factor 1-a | CCCTCCTCTTGGTCGTTTTG | ATGACACCAACAGCCACAGTTT |
| Reference 2 - H2A | Histone H2A | AGCGTTTAGCTGTGCTCCTTCC | TGACTCAATCGGTACCAGGGAAAC |
| HORVU.MOREX.r2.5HG0441650 | Zinc finger family protein | CCGATCATTTCGCCAGTAAT | GCCCACTTCTTTCAAGATCG |
| HORVU.MOREX.r2.2HG0129730 | Peroxidase | GCGGTCAAGAATCTCACCAT | GAAGTTGTAGAGCCGCTTCG |
| HORVU.MOREX.r2.1HG0056480 | ATP-dependent Clp protease ATP-binding subunit | CCGGTGATGACAGTGATACG | AGCCTTGATCGGCAAGTAGA |
| HORVU.MOREX.r2.2HG0094500 | CONSTANS-like zinc finger protein | GTACGTCGAGGACGCAGAG | CAAGATCAGCATGCCCAGTA |

**Supplementary Material 1.** The sequences of primers used for RT-qPCR analysis
