# Supplementary material for: Aluminum or Low pH – Which Is the Bigger Enemy of Barley? Transcriptome Analysis of Barley Root Meristem Under Al and Low pH Stress
Source: Front Genet. 2021 May 19;12:675260. doi: 10.3389/fgene.2021.675260 (PMC8244595; doi:10.3389/fgene.2021.675260)
Supplement: Supplementary file 1 [file Data_Sheet_1.zip › Table 14.DOCX]

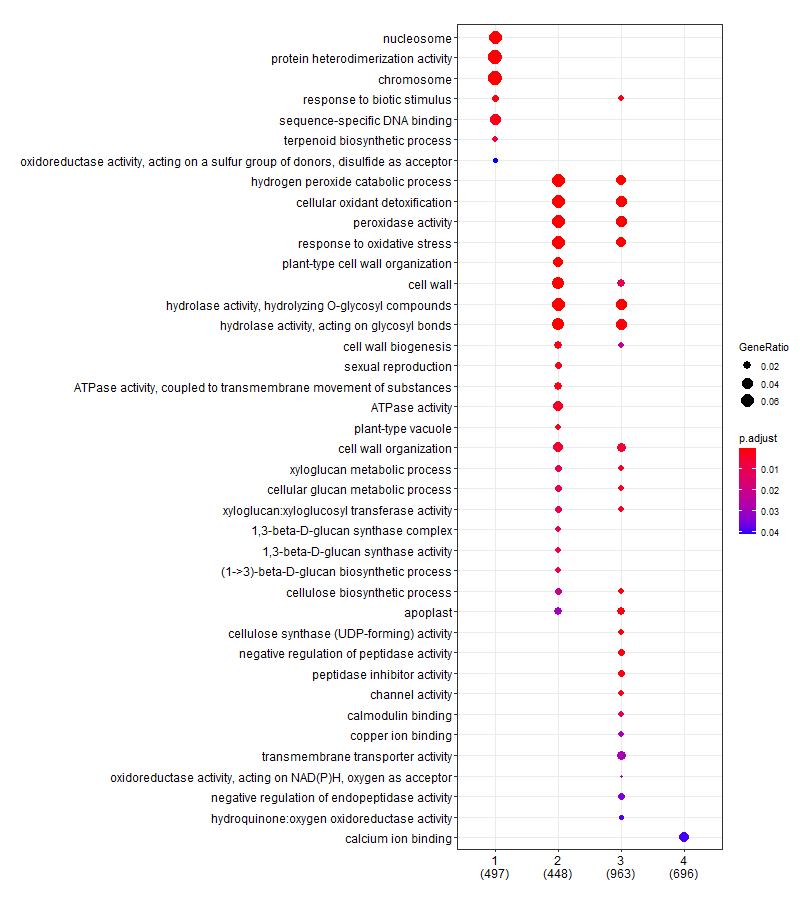


**Supplementary Material 14.** The comparison of GO terms over-representation analysis for detected clusters of genes (clusters 1-4, based on k-means clustering) with common expression patterns in the short-term experiment using hypergeometric test under α=0.05 with FDR adjusted *P*-value.
