# Supplementary material for: Aluminum or Low pH – Which Is the Bigger Enemy of Barley? Transcriptome Analysis of Barley Root Meristem Under Al and Low pH Stress
Source: Front Genet. 2021 May 19;12:675260. doi: 10.3389/fgene.2021.675260 (PMC8244595; doi:10.3389/fgene.2021.675260)
Supplement: Supplementary file 1 [file Data_Sheet_1.zip › Table 2.DOCX]

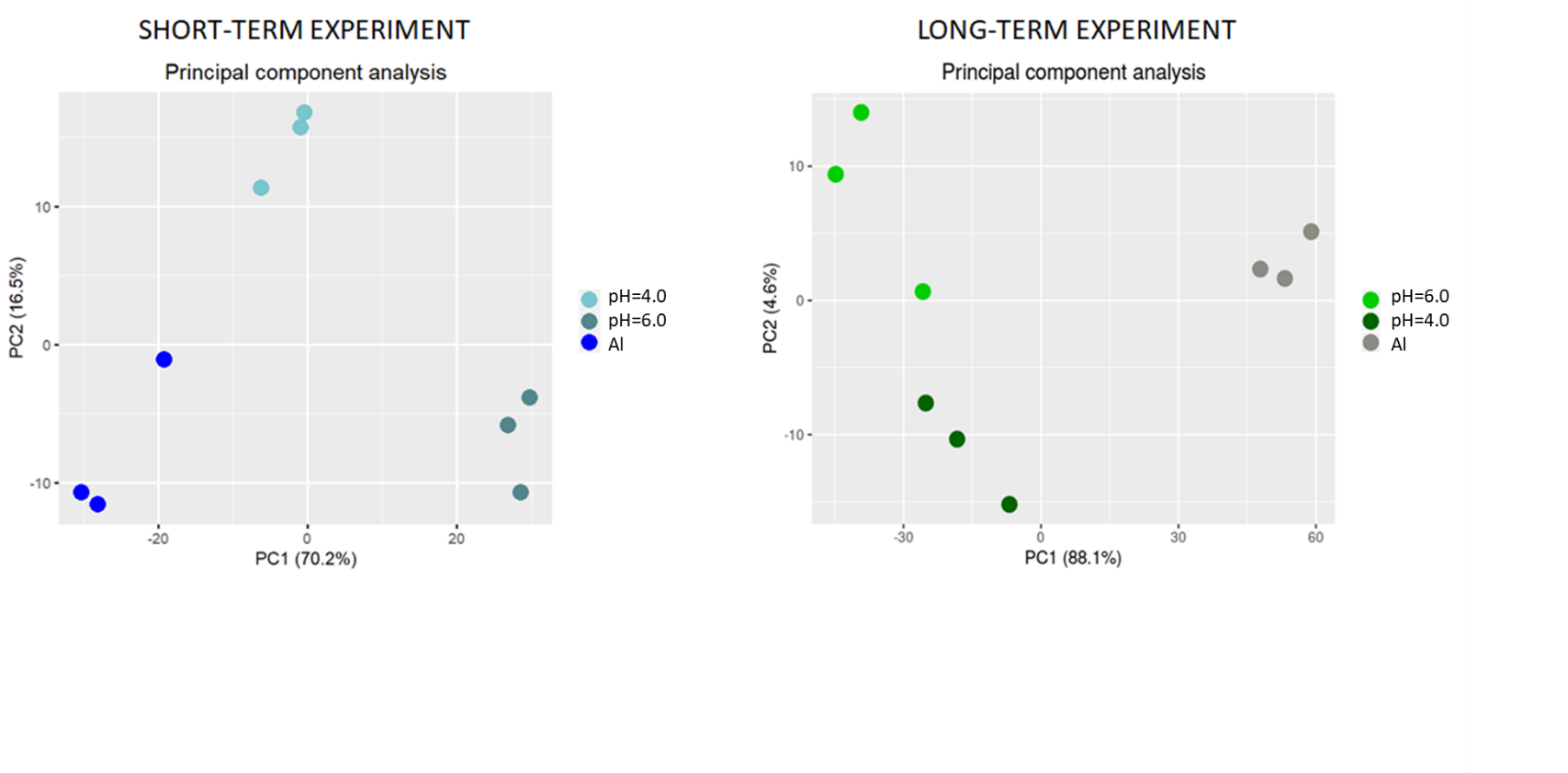


**Supplementary Material 2.** Principal component analysis (PCA) for transcriptomes of barley root meristem grown at pH=6.0, pH=4.0 and treated with 10 µM of bioavailable Al^3+^ in the short- and long-term experiments (one dot symbolizes one repetition).
