# Supplementary material for: Aluminum or Low pH – Which Is the Bigger Enemy of Barley? Transcriptome Analysis of Barley Root Meristem Under Al and Low pH Stress
Source: Front Genet. 2021 May 19;12:675260. doi: 10.3389/fgene.2021.675260 (PMC8244595; doi:10.3389/fgene.2021.675260)
Supplement: Supplementary file 1 [file Data_Sheet_1.zip › Table 3.DOCX]

1. **SHORT-TERM EXPERIMENT**

| GENE ID | Annotation | Log_2_FC | | | |
| --- | --- | --- | --- | --- | --- |
|  |  | pH=4 vs pH=6 | | Al vs pH=4 | |
|  |  | RNA-seq | qPCR | RNA-seq | qPCR |
| HORVU.MOREX.r2.5HG0441650 | Zinc finger family protein | 4,74* | 2,15* | -0,11 | 1,37 |
| HORVU.MOREX.r2.2HG0129730 | Peroxidase | 7,88* | 4,05* | -2,30* | -1,61* |
| HORVU.MOREX.r2.1HG0056480 | ATP-dependent Clp protease ATP-binding subunit | 5,96* | 2,25* | -0,88 | -0,97* |
| HORVU.MOREX.r2.2HG0094500 | CONSTANS-like zinc finger protein | -0,54 | -0,48* | -2,18* | -1,54* |

1. **LONG-TERM EXPERIMENT**

| GENE ID | Annotation | Log_2_FC | | | |
| --- | --- | --- | --- | --- | --- |
|  |  | pH=4 vs pH=6 | | Al vs pH=4 | |
|  |  | RNA-seq | qPCR | RNA-seq | qPCR |
| HORVU.MOREX.r2.5HG0441650 | Zinc finger family protein | 4,82* | 3,76* | -1,48 | -0,16 |
| HORVU.MOREX.r2.2HG0129730 | Peroxidase | 0,83 | 1,17 | -2,31* | -1,89* |
| HORVU.MOREX.r2.1HG0056480 | ATP-dependent Clp protease ATP-binding subunit | 6,16* | 3,57* | -6,25* | -1,05* |
| HORVU.MOREX.r2.2HG0094500 | CONSTANS-like zinc finger protein | -0,43 | 0,20 | -2,94* | -4,16* |

*statistically significant under α=0.05

**Supplementary Material 3.** Comparison of RNA-seq and qPCR expression profiles for four genes.

(A) Results for the short-term experiment. (B) Results for the long-term experiment.
